# Letter from Professor Powell

**Published:** 1849-01

**Authors:** 


### LETTER FROM PROFESSOR POWELL.

St. Genevieve, Mo., Oct. 16th, 1848.

*Dear Sir:—*

I concluded my geological and mineralogical tour this day; and having no company to night, I have resolved to devote a few hours to you. An opinion, briefly expressed as possible to be clearly understood, upon the principal objects that came under my notice, will not, I flatter myself, be entirely uninteresting to you. With this short preface, I will begin with the Iron Mountain.

This wonder in the natural history of our planet is not a mountain of iron, but a mountain two hundred feet high above the surrounding plane, covered with iron. The body of the mountain consists of slaty clay, which super-imposes sand stone. The covering of iron, judging from the excavations that have been made, is from a few inches to several feet in depth. This formation, you will perceive, precisely coincides with the geological system I had the pleasure of laying before you. At the base, sand stone, then slate and specular oxide of iron, and enough of it to supply several furnaces a thousand years. It will be rather difficult I think to assign an igneous origin to this mountain.

The Pilot Knob.—This is part of a chain of trap mountains, and is a greater wonder than the iron mountain. The inferior, three-fourths of it (from the best observations I could make,) consists of the micaceous oxide of iron and trap, which appear separately in large masses, though sometimes intimately blended. The iron possesses the structure of the trap, and about neither can any indications of stratification be seen. But super-imposing this amorphous trap and trappean iron, the latter, losing its former structure, becomes perfectly stratified. One thick stratum consists of slates of iron. Trappean matter is mixed in many places with the iron. This is more particularly the case with the last stratum, which is about fifteen feet thick. The dip is to the south—possibly a little west. Between the amorphous and stratified portions of this mountain, no other substance intervenes, nor are there any indications of a loss or lapse of time—the work was continued from one to the other. Now, sir, if we attribute the amorphous portion of this mountain to a plutonic or igneous agency, what shall we do with the stratified portion of it? Geologists have not in any instance, I believe, attributed stratification to an igneous agency. Could an aquatic agency have immediately followed the igneous? I think it could not; but suppose it could, why should the ferruginous action have continued? The truth is, Doctor, as you must perceive, this mountain is desperately in the way of the Plutonians; and I care not how rapidly the proprietors of it convert it into marketable iron, it will stand too long for their comfort, an evidence against them.

Mine La Mott.—At this place there is an extraordinary deposite of metalic ores, consisting of the sulphuret of lead, Bi-sulphurets of nickel and cobalt, and pyritous copper. These ores are contained in stratified limestone, which reposes on scinite, or concluding from the character of the masses, on a horn-blende granite. I have heard it asserted, that this mine was nearly exhausted. I do not believe that such an opinion will be very near the truth an hundred years hence.

Current River Copper District.—This copper, red porphyritic trap, has a length of about fifteen miles. This rock is slaty in its structure, and stratified in its masses; the strata, however, are not separable, but like some greenstones I have seen, are indicated by white lines. It passes into slates that resemble Novaculiti, and even into clay slate (what will the Plutonians do with this rock?) As a whole, in relation to the limestone that super-imposes it, it occupies the place of roof or argillaceous slate. The limestone is siliceous, and more than ordinarily soluble. There is no indication of any lapse of time between the conclusion of the porphyry and the beginning of the limestone. If this porphyry was produced in a state of igneous fluidity, no water could have been present for a very long time to produce the limestone. This is not all. If the porphyry is Plutonic, and the limestone Neptunic or aquatic, how could the copper lodes extend out of one into the other? The Plutonians may have a ready and plausible solution for these phenomena, but I confess I cannot anticipate it. There may be much copper in this porphyry; nevertheless, it is not the place for American capitalists, or miners. The former are too rare, and the latter not skilled enough in the art of mining. The prospects are tempting, but they will not fail to prove ruinous to men, or companies, of moderate capital.

Merrimac Copper Mines.—The copper here consists of the carbonates, the earthy brown, and red oxides, with a large per cent. of the oxide of iron. These ores are found in an ancient alluvion, with treccia, slaty clay, and usually large masses of a red sparry lime-rock. These materials repose, in interrupted strata, on the sides or slopes of hills. Science can no more aid here in finding the copper mines, than it can in the hunting of birds' nests; but the prospect once found, then science is of great service in determining the value of the prospect. In one respect, science is important. By it we can be sure where the ore is not. Several gentlemen there would have saved hundreds of dollars, had they possessed science sufficient to have known this. Any man who can dig for *float* lead, can obtain the copper here—hence this is the place for capitalists and miners. The ores are simple and manageable, and yet it seems that the requisite skill and science have not been obtained, as no copper has been produced.

The best of these mines will soon work out; they exist only in such pockets in the mountain's side as aquatic agency could not, or rather has not reached.

No man, therefore, is safe in paying a large price for a prospect; and if the proprietor thinks too highly of it to take a small one, he had better lease it. A flattering prospect may yield five hundred tons, and it may yield only five. It is true, that some estimate may be formed as to the probable richness of the mine, but it is also true that I saw no one in that district who appeared to know enough about geology to express an opinion on the subject. Scientific gentlemen may, nevertheless, be interested there; but the mining labor I saw, in several places, strongly contra-indicates it. When a man will *shaft* in the siliceous limestone of that district in search for copper, he has given proof that he is as ignorant of the geology of that copper, as he is of the Lunarians. Finally, I have never been more astonished, than at the abundant indications of mineral wealth in the State; which wealth, as yet, has scarcely commenced its development.

I must now leave you. Riding in the cold to-day, and the writing of these pages, have prepared me for rest; and I fear you will require rest by the time you have read this undigested mass of opinions, hurriedly thrown together.

With much esteem, yours very respectfully,

W. BYRD POWELL.

Dr. B. B. Brown.

